# Signaling Organizational Artificial Intelligence Adoption in Recruitment Materials: Role of Perceived Innovation Ability in Organizational Attractiveness

**DOI:** 10.3390/bs16030455

**Published:** 2026-03-19

**Authors:** Jialin Cheng, Shunhong Ji

**Affiliations:** 1 School of Economics and Management, Zhejiang Sci-Tech University, Hangzhou 310018, China; chengjl050520@zstu.edu.cn; 2 School of Business, Jishou University, No. 120 Renmin South Road, Jishou 416000, China

**Keywords:** AI-adoption signaling, perceived innovation ability, organizational attractiveness, AI self-efficacy

## Abstract

Although previous studies have examined factors influencing organizational appeal, how AI-adoption signals influence prospective applicants remains unclear. Building on signaling theory, this study explores whether, when, and how organizations’ AI-adoption signals enhance their attractiveness to potential applicants. Two experiments were conducted to test the hypothesized model. Study 1 (N = 145) employed a scenario-based design to compare organizational attractiveness between AI-adoption signal and no-signal conditions, confirming that AI-adoption signals are significantly positively associated with organizational attractiveness. Study 2 (N = 240) recruited active job seekers and validated a moderated mediation model: perceived innovation ability mediates the positive association between AI-adoption signals and organizational attractiveness, especially among job seekers with high AI self-efficacy. By conceptualizing AI adoption as an organizational signal, this research extends signaling theory to the context of technology-infused recruitment and offers practical insights for designing more effective recruitment strategies in the digital era.

## 1. Introduction

With the rapid advancement of digital transformation, the application of artificial intelligence (AI) is expanding beyond production, manufacturing, and customer service to include human resource management, with recruitment serving as a prominent example (Basu et al., 2023; Black & van Esch, 2020; Chowdhury et al., 2023; Pereira et al., 2023). In China, the rapid advancement of AI technologies has been strongly supported by national policies such as the New Generation Artificial Intelligence Development Plan (The State Council of the People’s Republic of China, 2017), which encourages enterprises to integrate AI into business operations, including recruitment. Chinese firms have increasingly adopted AI-based recruitment tools for tasks such as résumé screening, applicant interviews, and talent analytics (Ling et al., 2025; Min et al., 2024; N. Wang et al., 2025; J. X. Wang et al., 2025; Zheng et al., 2024). This context makes China a particularly relevant setting for examining how job seekers interpret organizational signals of AI adoption.

Beyond transforming recruiting practices, AI use may also implicitly signal organizational values or traits to prospective applicants, potentially shaping their perceptions and evaluations of the firm’s overall attractiveness. Prior research has predominantly investigated factors such as compensation, work practices, and corporate social responsibility in determining organizational appeal (Belinda et al., 2018; Connelly et al., 2011, 2024; Jakob et al., 2022; Kröll et al., 2021); however, how explicit statements of AI adoption function as a signal to potential applicants—particularly in an era of increasingly ubiquitous AI usage—remains unclear. Consequently, this work aims to answer the following research questions: Is a firm’s AI-adoption signaling associated with organizational attractiveness? If so, which underlying factors may help explain this association, and which factors moderate this association? Signaling theory offers a robust theoretical foundation for this study. In markets characterized by information asymmetry—wherein, for example, job seekers cannot fully assess a company’s true qualities—organizations must use observable “signals” to communicate intangible attributes (Connelly et al., 2024; Highhouse et al., 1999; Spence, 1973). We argue that clearly highlighting an organization’s AI use in recruitment materials may function as a distinctive and powerful signal of the organization’s commitment to adopting advanced technologies while implicitly conveying key traits, such as an innovative ability (Bahoo et al., 2023; Lin et al., 2024; Mariani et al., 2023).

This study posits that a firm’s AI-adoption signaling may help enhance organizational attractiveness, with perceived innovation ability being a significant mediating variable. Previous research has found innovation ability to be a critical antecedent of employer attractiveness (Davies et al., 2025; Maier et al., 2022; Sommer et al., 2017). However, the literature has yet to clarify which specific organizational technology signals may shape job seekers’ perceptions of an organization’s innovation ability, especially in the context of digital transformation, where AI technology has become a core organizational characteristic (Davies et al., 2025; Maier et al., 2022; Sommer et al., 2017). This study, thus, positions perceived innovation ability as a critical factor connecting organizational digital signals and employer attractiveness, offering insights into how technological signaling may influence job seekers. Further, AI self-efficacy, which refers to an individual’s confidence in their ability to accomplish AI-related tasks, is proposed as a moderating variable (Dong et al., 2024; Hong, 2022). Job seekers with higher AI self-efficacy may be more likely to perceive firms that signal AI adoption as possessing greater innovative capabilities, subsequently increasing the firm’s attractiveness.

This study conceptualizes AI-adoption signaling as a distinct employer signal, thereby broadening the traditional boundaries of recruitment signals (Aronson et al., 2025; Connelly et al., 2011, 2024; D. Phillips et al., 2024; Waples & Brachle, 2020) and systematically examining the role of AI-related signals in attracting potential applicants. Additionally, this study identifies “perceived innovation ability” as a significant mediator between organizational AI-adoption signals and organizational attractiveness, thus providing valuable insights into the association between AI-adoption signaling and organizational attractiveness. Furthermore, this study suggests that potential job seekers’ AI self-efficacy moderates this association, highlighting the significance of individual characteristics in the signal interpretation process and underscoring the importance of signal–individual fit for future studies. Finally, this study contributes to the integration of the literature on AI technology applications and organizational attractiveness, offering empirical support for theoretical work on the use of AI in human resource management (HRM) and novel insights into organizational signaling in the digital era.

## 2. Research Model and Hypothesis Development

### 2.1. Signaling of Organizational AI Adoption and Organizational Attractiveness

Signaling theory holds that under information asymmetry, the better-informed party utilizes observable signals to convey information about otherwise unobservable attributes to the less-informed party, which then interprets these signals to assess the former side’s true value, thereby reducing decision-making risk (Connelly et al., 2011; Spence, 2002; Taj, 2016). Within the job-search context, job seekers primarily seek employment from organizations that they perceive as able to fulfill their needs. However, a firm’s true capabilities are not directly observable; for example, job seekers cannot readily know whether the firm will implement layoffs or has the capacity to honor its compensation commitments (Boswell et al., 2003; Goldberg & Willham, 2024; Merriman et al., 2025). In this regard, AI adoption, as an observable signal, becomes the basis on which job seekers evaluate employers; specifically, they may infer that AI adoption correlates with desirable firm attributes and, therefore, perceive the firm as more attractive.

AI refers to intelligent machines and computer-based systems that learn, respond, and perform varied human-like tasks (Xu et al., 2024). Organizational AI adoption is defined as the extent to which an organization has implemented AI technologies into its internal processes, operations, or products (Lin et al., 2024). In contrast, AI-adoption signaling refers to the intentional and explicit mention of an organization’s use of AI within its externally facing recruitment materials. Compared with abstract claims such as “the company values innovation” or “the company values high efficiency,” “the company adopts AI” is more concrete and verifiable (Smith et al., 2025), enabling job seekers to form a low-cost preliminary view of a company’s attributes and identify high-quality employers.

In the hiring market, companies that can invest in AI adoption and deployment typically possess stronger financial resources and a more forward-looking strategic vision (Johnston & Cortez, 2023; Lin et al., 2024), sharply distinguishing them from firms that are less competitive and less resilient. Moreover, job seekers equate “AI adoption” with being “at the industry’s technological frontier,” often assuming that such companies can better weather market volatility and are more innovative than others (Liu, 2025; Siddik et al., 2025; Tekic & Füller, 2023). Consequently, job seekers are drawn to employers that send strong signals across job advertisements (Sommer et al., 2017). Therefore, disclosing “AI adoption” in job postings could help companies positively signal to job seekers, mitigate information asymmetry in hiring, and ultimately increase their appeal to candidates. Accordingly, the following hypothesis is proposed:

**Hypothesis** **1 (H1).** *Potential applicants perceive firms with AI-adoption signals (versus no AI-adoption signals) as more attractive.*

### 2.2. Mediating Role of Perceived Innovation Ability

Rooted in the core propositions of signaling theory (Connelly et al., 2011; Spence, 1973), this study posits that organizational AI-adoption signaling is positively associated with organizational attractiveness, explained by the role of perceived innovation ability. Signaling theory emphasizes that in an information-asymmetric labor market, observable organizational signals must have a material association with unobservable organizational attributes to enable effective inference by signal receivers (Connelly et al., 2011, 2024; Taj, 2016). Perceived innovation ability is hypothesized as the central mediating factor linking AI-adoption signals and organizational attractiveness because it conforms to the core logic of signaling theory, unlike other potential explanatory pathways (e.g., organizational prestige, job security).

AI, as a cutting-edge interdisciplinary technology integrating machine learning, natural language processing, and automated reasoning (Bankins et al., 2024; Fan et al., 2023; Tang et al., 2023), is a paradigmatic symbol of technological innovation (Füller et al., 2024; Jeong & Jeong, 2025). Explicitly mentioning AI adoption in recruitment materials acts as a diagnostic signal (Connelly et al., 2024) for job seekers to judge the firm’s innovation orientation: unlike abstract and unverifiable claims of “valuing innovation,” AI-adoption signaling is a concrete, action-oriented way for employers to provide tangible evidence of the organization’s engagement in innovative technological practice (Smith et al., 2025). In line with signaling theory’s inference mechanism, job seekers, as the information-disadvantaged party, rely on this diagnostic signal to decode the organization’s latent innovation ability. They associate the firm’s active adoption of AI with a willingness to break traditional operational models, experiment with novel technologies, and pursue technological and business innovation (Allal-Cherif et al., 2023; Liu, 2025; Robertson et al., 2025).

While other factors, such as perceived organizational prestige and job security, may also explain the attractiveness of AI-adopting firms, we argue that perceived innovation ability is theoretically distinct and particularly salient in the context of digital transformation. Unlike perceived organizational prestige, which may stem from brand reputation or corporate social responsibility (Rodrigo et al., 2019; Wayne & Casper, 2012), innovation ability is directly inferred from technological signals and reflects the firm’s future-oriented capacity. Similarly, although AI adoption may imply job security through financial stability or career development through skill upgrading (Grolleau et al., 2022; Miao et al., 2023), these perceptions are likely downstream consequences of perceived innovativeness rather than parallel mediators.

Furthermore, signaling theory emphasizes that credible signals are often costly to imitate (Spence, 1973, 2002)—the implementation and sustainable operation of AI technology require substantial diverse resource inputs, including financial capital for technological procurement and R&D, professional technical talents for system optimization, and organizational support for workflow reconstruction and cultural adaptation (Fountaine et al., 2019; Uren & Edwards, 2023). For job seekers, the costliness of AI adoption makes this signal highly credible: it signals not only the organization’s innovative intention but also its capability to bear the high costs of innovation (Broekhuizen et al., 2023; Robertson et al., 2025). Moreover, job seekers infer that organizations capable of investing in and deploying AI possess the resource endowments and strategic vision to support continuous innovation in R&D, business transformation, and other fields (Uren & Edwards, 2023). Accordingly, they perceive such firms as having higher innovation ability than those that do not signal AI adoption. Accordingly, we posit the following hypothesis:

**Hypothesis** **2 (H2).** *AI-adoption signaling is positively associated with firms’ perceived innovation ability.*

As proposed in Hypothesis 2, credible AI-adoption signaling enables job seekers to form positive perceptions of organizational innovation ability. Moreover, perceived innovation ability, as an important outcome of signal interpretation, further drives job seekers to evaluate organizational attractiveness positively, because this inferred attribute responds to the core information demands of job seekers in the information-asymmetric labor market: job seekers are not only concerned about the explicit conditions of a position; more importantly, they need to judge the sustainable development potential and long-term value of the organization through observable signals (Highhouse et al., 1999; Connelly et al., 2011), and perceived innovation ability is just the key attribute that reflects an organization’s potential for sustainable development.

According to signaling theory, perceived innovation ability implicitly transmits three types of positive information about the organization to job seekers, all of which directly enhance the organization’s attractiveness in the job seekers’ perceptions. First, perceived innovation ability signals the resource advantage of the organization over its competitors. Job seekers infer that organizations with strong innovation ability have sufficient financial capital, professional technical talents, and organizational systems to support continuous innovation activities (Camisón & Villar-López, 2014; Hervas-Oliver & Sempere-Ripoll, 2015; Sommer et al., 2017). These resource endowments suggest that the organization may be better able to withstand market risks (Jiménez-Jiménez & Sanz-Valle, 2011), which alleviates job seekers’ concerns about employment risk while encouraging them to evaluate the organization positively.

Second, perceived innovation ability signals the organization’s development vitality. In the digital era, innovation ability is the core driving force of organizational sustainable development (Tekic & Füller, 2023; Luo & Bhattacharya, 2006). Job seekers who interpret the organization’s strong innovation ability as a signal of strong growth potential are more likely to believe that working in such an organization can provide more long-term career development opportunities, making them more willing to join the organization.

Third, perceived innovation ability signals organizational value orientation. Job seekers perceive organizations with strong innovation ability as willing to subvert the status quo, encourage exploration and experimentation, and reward employees’ innovative behavior (Sommer et al., 2017; Grolleau et al., 2022). This value orientation aligns with the career development expectations of most job seekers, especially those with high AI self-efficacy, and, thus, likely enhances organizational attractiveness. Moreover, prior research suggests that perceived innovation ability is a critical antecedent of organizational attractiveness (Davies et al., 2025; Maier et al., 2022; Sommer et al., 2017). Accordingly, we posit the following hypothesis:

**Hypothesis** **3 (H3).** *Signaling AI adoption is positively associated with organizational attractiveness through the perceived innovation ability of firms.*

### 2.3. AI Self-Efficacy’s Moderating Role

According to signaling theory, under information asymmetry, senders convey otherwise unobservable attributes through specific observable signals, while receivers interpret those signals using their knowledge and experience to infer the sender’s covert characteristics (Branzei et al., 2004; Connelly et al., 2011; Kirmani & Rao, 2000). Within hiring contexts, a firm’s AI adoption functions as such a signal, with job seekers’ AI self-efficacy directly shaping how they interpret it (Highhouse et al., 2007). The present study posits that high AI self-efficacy enables job seekers to more easily recognize a firm’s innovative ability from its AI-adoption signal.

AI self-efficacy is the confidence employees have in their ability to complete AI-related tasks (Bandura, 1977; Dong et al., 2024). Job seekers with high AI self-efficacy are confident in their ability to learn and use AI (Dong et al., 2024; He et al., 2025). That confidence typically rests on their existing AI-related knowledge, skills, or prior successes (Dong et al., 2024; He et al., 2025). Accordingly, they possess rich knowledge for interpreting a firm’s AI-adoption signal. For example, when encountering descriptions such as “enterprise AI adoption,” they are more likely to comprehend the technical aspects and implementation difficulties (Bankins et al., 2024; Tang et al., 2023). They also recognize that successfully deploying AI requires sustained R&D investment, substantial funding, and workflow redesign—factors that indicate the firm’s innovation ability (Füller et al., 2024; Jeong & Jeong, 2025). By contrast, job seekers low in AI self-efficacy typically lack related technical knowledge (Dong et al., 2024; He et al., 2025). Even if a job posting states “the company uses AI,” they are less likely to understand the technical substance and value of this statement. They are more likely to interpret the signal superficially or negatively (Hong, 2022), potentially inferring that the firm has low innovation ability.

Second, signaling theory posits that when a signal conveys the potential fulfillment of the receiver’s needs, they are more likely to evaluate the sender positively (Connelly et al., 2011; Ehrhart & Ziegert, 2005). Job seekers with high AI self-efficacy typically regard AI competence as one of their core competitive advantages (Jin et al., 2025; Kim & Kim, 2024) and generally choose employers that recognize and utilize their AI skills. They interpret a firm’s AI adoption as a signal that it values AI technology and appreciates its benefits, aligning with their skill set and career expectations; this perceived fit can motivate them to explore the firm’s strengths (Connelly et al., 2011), with innovative ability being one of the most readily associated with AI-adopting firms (Füller et al., 2024; Jeong & Jeong, 2025). By contrast, job seekers with low AI self-efficacy are less likely to perceive a firm’s AI adoption as matching their capabilities and career expectations; owing to their limited confidence in completing AI-related tasks, they may worry about adapting to the firm’s technical environment (Hong, 2022; Kim & Kim, 2024). Even when they recognize that a firm adopts AI, they may focus instead on whether the job will require grappling with complex AI tools (Hong, 2022; Kim & Kim, 2024), making the firm’s innovative ability more difficult to perceive. Accordingly, we posit the following hypothesis:

**Hypothesis** **4 (H4).** *The positive association between signaling AI adoption and perceived innovation ability is stronger for job seekers with high AI self-efficacy than for those with low AI self-efficacy.*

**Figure 1:**
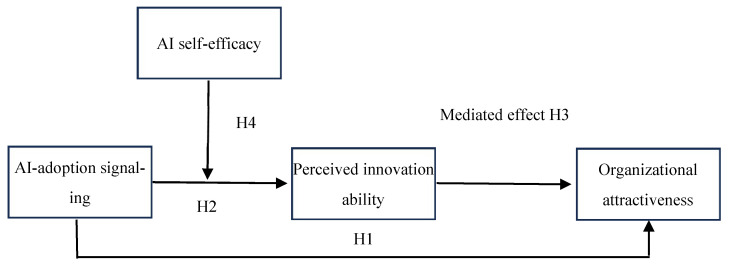
Conceptual model of the present research.

Combining the analyses in previous sections, we posit the following moderated mediation hypothesis:

**Hypothesis** **5 (H5).** *AI self-efficacy moderates the indirect association between AI-adoption signaling and organizational attractiveness through perceived innovation ability, such that the association is stronger when AI self-efficacy is higher. Figure 1 illustrates this study’s theoretical model.*

## 3. Study 1: Materials and Methods

### 3.1. Participants

We recruited 145 participants through Credamo (https://www.credamo.com)—a platform that facilitates online participant recruitment and provides high-quality data support for experiments and surveys (Cai et al., 2023; Dong et al., 2024). A priori power analysis conducted using G*Power software 3.1 (Faul et al., 2007) revealed that the required sample size was N = 128, based on the preconditions of achieving a large effect size (f^2^ = 0.50) and 80% statistical power. In Study 1, we recruited 145 participants, thus satisfying this criterion.

Each participant who completed our experiment received 2 RMB as compensation. The final comprised 91 women (62.8%) and 54 men (37.2%). Only one participant (0.7%) was under 20 years old; most (89, 61.4%) were aged 20–29, while 48 (33.1%) were aged 30–40, followed by those aged 41–50 (5, 3.4%) and above 50 (2, 1.4%). Most participants held a bachelor’s degree (109, 75.2%), while others had a master’s degree (27, 18.6%), followed by those who had completed college education (7, 4.8%) and high school/polytechnic school education (2, 1.4%). Some participants had no work experience (15, 10.3%), but most had worked for 1–10 years (111, 76.6%), while others had worked for 11–20 years (15, 10.3%), and a few had worked for over 20 years (4, 2.8%).

### 3.2. Procedure and Experimental Design

We manipulated AI-adoption signaling by asking participants to imagine that they are currently seeking a job. Participants were randomly assigned to one of two experimental conditions: the signal condition, in which participants encountered a job posting that included a statement about the company’s use of AI, or the no-signal condition, in which participants encountered the same posting but with no AI-adoption statement.

The job postings were adapted from real-world recruitment advertisements collected from Zhaopin, a major Chinese job platform (https://www.zhaopin.com/). We intentionally used generic job posting content, excluding industry-specific terminology and unique corporate identifiers, to reduce systematic variation unrelated to the inclusion or exclusion of AI-related wording across conditions. This approach follows established recommendations for experimental manipulation in recruitment research, where the goal is to manipulate a single signal while holding all other factors constant (Aguinis & Bradley, 2014).

To avoid potential biases related to real company names or industries, we used a fictitious company name, “QXC,” in all job postings. This approach ensures that participants’ responses are based solely on the manipulated content (i.e., the presence or absence of AI-related signals) rather than on prior knowledge or associations with existing organizations. Participants exposed to the signal condition encountered the following text:

*QXC is rapidly expanding and welcomes highly motivated*, *results-driven*, *responsible*, *and energetic individuals to join us across a variety of roles. We are actively integrating AI technologies to continuously strengthen our core competitiveness. For instance*, *our operations team leverages AI to ensure efficient resource allocation*, *facilitate seamless collaboration*, *and deliver exceptional services. Further*, *job responsibilities will be tailored to your experience. For detailed information*, *please refer to the job listings on our official website. We offer a competitive salary*, *with compensation determined according to individual skills and experience. Additionally*, *QXC provides outstanding employee benefits. If you are interested*, *please call 0571-8366239 or visit the QXC official website to learn more about our current openings.*

Participants exposed to the no-signal condition encountered the following text:

*QXC is rapidly expanding and invites highly motivated*, *results-driven*, *responsible*, *and energetic individuals to join us across a variety of roles. Further*, *job responsibilities will be tailored to your experience. For detailed information*, *please refer to the job listings on our official website. We offer a competitive salary*, *with compensation determined according to individual skills and experience. Additionally*, *QXC provides outstanding employee benefits. If you are interested*, *please call 0571-8366239 or visit the QXC official website to learn more about our current openings.*

Overall, 145 participants were randomly assigned to the signal condition or the no-signal condition. Accordingly, after reading one of the above two job postings, participants completed scales measuring AI-adoption signaling and organizational attractiveness. Finally, participants provided their demographic information.

### 3.3. Measures

Unless otherwise specified, all measures employed herein were rated on a seven-point Likert-type scale ranging from “1 = Strongly disagree” to “7 = Strongly agree.”

Manipulation Check. We checked the manipulation of AI-adoption signaling using a three-item scale adapted from Cheng et al. (2023). A sample item is as follows: “This company has been involved in AI technology adoption.” (α = 0.951).

Organizational attractiveness. We measured organizational attractiveness using five items adapted from Highhouse et al. (2003). A sample item is as follows: “For me, this company would be a suitable place to work.” (α = 0.945)

## 4. Study 1: Results

### 4.1. Manipulation Check

First, we conducted a manipulation check for the AI-adoption signaling. All statistical analyses were performed using IBM SPSS Statistics (Version 27.0). To verify the success of this manipulation, an independent-samples t-test was conducted to compare participants’ perceived agreement that the company had adopted AI between the signal and no-signal conditions. Participants in the signal condition reported significantly stronger agreement that the company had adopted AI (N = 73, M = 6.30, SD = 0.50) compared to those in the no-signal condition (N = 72, M = 4.06, SD = 1.64, t [84.14] = 11.13, *p* < 0.001, Cohen’s d = 1.85), indicating that the manipulation was successful.

### 4.2. Hypothesis Tests

The independent samples t-test (analyzed using IBM SPSS Statistics, Version 27.0) revealed that participants exposed to the AI signal reported higher attractiveness ratings, with a mean firm attractiveness rating of 6.05 (SD = 0.57) among job seekers, significantly surpassing the mean score of 5.15 (SD = 1.48) when the advertisement did not reference AI adoption. The detailed statistical results were as follows: t (91.56) = 4.83, *p* < 0.001, and Cohen’s d = 0.80.

Moreover, the regression results analyzed using IBM SPSS Statistics (Version 27.0) demonstrated that AI-adoption signaling exhibited a significantly positive association with organizational attractiveness (b = 0.46, SE = 0.05, *p* < 0.001, R^2^ = 0.42, F = 19.72), after controlling for gender, age, education, and work experience. In the model without control variables, AI-adoption signaling also has a significant positive association with organizational attractiveness (b = 0.46, SE = 0.05, *p* < 0.001, R^2^ = 0.40, F = 95.04). Thus, H1 was supported.

### 4.3. Discussion of Findings

Study 1 provides preliminary experimental evidence that AI-adoption signals may enhance organizational attractiveness, thereby supporting the basic phenomenon focused on in this research. However, Study 1 did not examine how this effect occurs, nor did it set the sample as real job seekers. To address these issues, Study 2 extends the research findings in the following aspects: (a) recruiting real job seekers to improve external validity; (b) testing the mediating role of perceived innovation ability; (c) examining the moderating role of AI self-efficacy. These two studies follow a logical progression from establishing the potential effect to explaining its internal mechanism and boundary conditions.

## 5. Study 2: Materials and Methods

### 5.1. Participants

We recruited 240 actual job seekers through the Credamo platform (https://www.credamo.com). This sample size was selected to ensure adequate statistical power. A priori power analysis using G*Power (Faul et al., 2009) indicated that, to detect a large effect size (f^2^ = 0.50) with 0.90 power at α = 0.05, a minimum of approximately 210 participants was required. To account for potential exclusions and ensure robustness, we recruited 240 actual job seekers. To ensure eligibility, we asked each participant the following question: “Are you currently looking for a job?” Those who answered “yes” were allowed to participate in the experiment, while those who answered “no” were excluded. Participants who completed the experiment received a monetary reward of 3 RMB.

The final sample comprised 136 women (56.67%) and 104 men (43.33%). Only 2 participants were aged 20 or younger (0.83%), 123 were 21–30 (51.25%), 94 were 31–40 (39.17%), 11 were 41–50 (4.58%), and 10 were over 50 (4.17%). Seven participants had completed senior high school or technical secondary school (2.92%), 20 had an associate degree (8.33%), 175 had a bachelor’s degree (72.92%), and 38 had a master’s degree (15.83%). Most participants had 2–10 years (161, 67.08%) of work experience, followed by 37 with one year or less (15.42%), 32 with 11–20 years (13.33%), and 10 with over 20 years (4.17%).

### 5.2. Procedure and Experimental Design

Before commencing the experiment, participants were asked to treat the upcoming job advertisement as if it were posted by a company that they had recently encountered. The experimental materials were identical to those utilized in Study 1. To manipulate the signaling of organizational AI adoption, the following statement was either included or omitted from the job posting: “We are actively integrating AI technologies to continuously strengthen our core competitiveness. For instance, our operations team leverages AI to ensure efficient resource allocation, facilitate seamless collaboration, and deliver exceptional services.”

Study 2 employed a single-factor between-subjects design, with participants randomly assigned to either the control group, wherein the job posting did not mention company AI adoption, or the experimental group, wherein the posting highlighted the company’s AI use. Participants were asked to read the job advertisement carefully, with no time limit imposed. Thereafter, they completed scales measuring perceived AI-adopting signaling, innovation ability, organizational attractiveness, and AI self-efficacy. Finally, they provided their demographic information, such as gender and age, after which they received a thank-you note for their participation.

### 5.3. Measures

Unless otherwise specified, all measures were rated on a seven-point Likert-type scale ranging from “1 = Strongly disagree” to “7 = Strongly agree.” Please see Appendix A. 

Manipulation Check. We checked the manipulation of AI-adopting signaling using a three-item scale adapted from Cheng et al. (2023). A sample item is as follows: “This company has been involved in AI technology adoption.” (α = 0.95).

AI self-efficacy. We measured AI self-efficacy using three items adapted from Dong et al. (2024). A sample item is as follows: “I am confident about my ability to perform my job duties related to the AI/working with the AI.” (α = 0.76).

Perceived innovation ability. We measured perceived innovation ability using Luo and Bhattacharya’s (2006) three-item scale. A sample item is as follows: “I believe that this company is highly innovative.” (α = 0.95).

Organizational attractiveness. We measured organizational attractiveness using five items adapted from Highhouse et al. (2003). A sample item is as follows: “For me, this company would be a suitable place to work.” (α = 0.96).

## 6. Study 2: Results

Correlation analysis using IBM SPSS Statistics (Version 27.0) revealed that AI-adoption signaling was positively associated with organizational attractiveness (r = 0.61, *p* < 0.001) and perceived innovation ability (r = 0.73, *p* < 0.001). Additionally, perceived innovation ability was positively associated with organizational attractiveness (r = 0.81, *p* < 0.001). Furthermore, the variance inflation factor (VIF) values for AI-adoption signaling, perceived innovation ability, and AI self-efficacy were 2.17, 2.18, and 1.01, respectively. Evidently, all VIF values were below 3, indicating that multicollinearity was not a concern in our models (Akinwande et al., 2015).

### 6.1. Confirmatory Factor Analysis

This study performed confirmatory factor analysis using Mplus Version 7. The results in Table 1 indicate that the four-factor model (AI-adoption signals, perceived innovation ability, organizational attractiveness, and AI self-efficacy) exhibited the most optimal fit (χ^2^/df = 1.75; CFI = 0.98; TLI = 0.98; RMSEA = 0.06; SRMR = 0.02). indicating satisfactory discriminant validity.

### 6.2. Manipulation Check

First, we checked the manipulation of AI-adoption signaling. Independent samples *t*-tests via IBM SPSS Statistics (Version 27.0) showed that participants in the signal condition reported significantly stronger agreement that the company had adopted AI (N = 120, M = 6.16, SD = 0.49) than those in no-signal condition (N = 120, M = 3.59, SD = 1.66, t [139.75] = 16.22, *p* < 0.001, Cohen’s d = 2.10). These results confirm that we successfully manipulated the AI-adoption signal.

### 6.3. Hypothesis Tests

The independent samples t-test via SPSS (version 27.0) revealed that AI-adoption signaling significantly enhanced organizational attractiveness. Specifically, participants in the signaling condition reported higher attractiveness, with a mean rating of 6.07 (SD = 0.69), significantly surpassing the mean score of 4.99 (SD = 1.71) in the non-signal group. The detailed statistical results were as follows: t (156.90) = 6.41, *p* < 0.001, and Cohen’s d = 0.82. An independent samples t-test conducted via SPSS (Version 27.0) indicated that participants in the signal condition reported significantly higher perceived innovation ability (M = 5.84, SD = 0.64) than those in the control condition (M = 4.45, SD = 1.75; t [150.40] = 8.20, *p* < 0.001, Cohen’s d = 1.06).

Next, we performed regression analyses using SPSS 27.0 to test Hypotheses 1, 2, and 4 (Table 2). The results for Model 5 demonstrated that AI-adoption signaling exhibited a significantly positive association with organizational attractiveness (b = 0.48, SE = 0.04, *p* < 0.001). Thus, these results further supported H1. The results for Model 2 further revealed that AI-adoption signaling was positively associated with perceived innovation ability (b = 0.61, SE = 0.04, *p* < 0.001). Collectively, these findings support H2. The Model 3 results reveal that AI self-efficacy moderated the association between AI-adoption signals and perceived innovation ability (b = 0.14, SE = 0.04, *p* < 0.01). Thus, these results further supported H4.

This study employed Model 4 of the PROCESS macro (Hayes, 2013; Hayes & Preacher, 2014; Preacher & Hayes, 2004) in SPSS version 27 to test the mediating role of perceived innovation ability. After controlling for age, gender, educational level, and years of work experience, the analysis, based on 5000 bootstrap samples, revealed that perceived innovation ability significantly mediated the association between AI-adoption signaling and organizational attractiveness (estimate = 0.46, SE = 0.06, 95% confidence interval [CI] = [0.35, 0.58]). Thus, H3 was supported.

**Figure 2:**
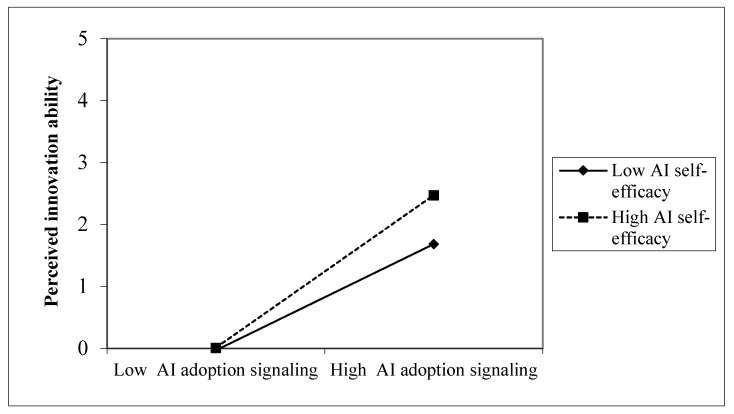
Artificial Intelligence (AI) Self-efficacy Moderates the Relationship between AI Adoption Signaling and Perceived Innovation Ability.

This study employed the bootstrap method (Hayes, 2013; Hayes & Preacher, 2014; Preacher & Hayes, 2004), as implemented in Mplus (Version7), to test the moderating effect of AI self-efficacy on the relationship between AI-adoption signaling and perceived innovation ability. The results revealed a significant moderating effect, as the 95% CI did not include zero (estimate = 0.14, SE = 0.04, 95% CI = [0.05, 0.22]; Figure 2). Moreover, the association between AI-adoption signaling and perceived innovation ability was stronger at higher (+1 SD) AI self-efficacy levels (estimate = 0.69, SE = 0.06, 95% CI = [0.58, 0.81]) compared to lower (−1 SD) levels (estimate = 0.48, SE = 0.08, 95% CI = [0.33, 0.64]). Additionally, the difference between those slopes was significant (estimate = 0.21, SE = 0.10, 95% CI = [0.01, 0.41]). Thus, H4 was further supported.

We used Mplus (Version7) to conduct a bootstrap analysis (Hayes, 2013) with 5000 samples to test the moderated mediation effect (moderated mediation model equivalent to PROCESS Model 7) (Edwards & Lambert, 2007; Preacher et al., 2007). When AI self-efficacy was high, the indirect association between AI-adoption signaling and organizational attractiveness through perceived innovation ability was positive (effect = 0.52), with a 95% CI excluding zero (95% CIs [0.39, 0.65]); when AI self-efficacy was low, AI-adoption signaling’s indirect association with organizational attractiveness through perceived innovation ability was positive (effect = 0.36), with a 95% CI excluding zero (95% CIs [0.22, 0.50]); the two indirect associations differed significantly (effect = 0.16, 95% CIs [0.01, 0.31]). Hence, H5 was supported.

### 6.4. Discussion of Findings

Study 2 more deeply investigated the association between AI-adoption signaling and organizational attractiveness. Its results further supported H1 by demonstrating that AI-adoption signaling has a significantly positive association with organizational attractiveness. Additionally, Study 2 revealed that perceived innovation ability mediates the association between AI-adoption signaling and organizational attractiveness, thereby substantiating H3. Extending Study 1, Study 2 offered robust evidence for AI self-efficacy’s moderating role in the aforementioned association. Specifically, when job seekers possess higher AI self-efficacy levels, the positive association between AI-adoption signaling and perceived innovation ability becomes more pronounced, thus supporting H4. Finally, Study 2 examined the presence of a moderated mediation effect, indicating that job seekers’ AI self-efficacy significantly moderated perceived innovation ability’s mediating role in the association between AI-adoption signaling and organizational attractiveness, thus supporting H5.

## 7. Discussion

### 7.1. General Discussion

Drawing on signaling theory (Connelly et al., 2024; Spence, 1973), this work reveals that highlighting an organization’s AI use in recruitment materials may significantly enhance its appeal to prospective job seekers. Job seekers’ perceptions of the organization’s innovative abilities mediate this association. This study extends the antecedent framework of organizational attractiveness by introducing AI adoption as a novel digital technological signal, moving beyond the traditional focus on non-technological antecedents such as organizational brand types, corporate image, corporate social responsibility, and work practices (Belinda et al., 2018; Jakob et al., 2022; Kröll et al., 2021; L. S. Wu & Dineen, 2023; Younis & Hammad, 2021). In the context of digital transformation, where AI has become a core organizational characteristic (Bahoo et al., 2023; Basu et al., 2023; Füller et al., 2024), this finding provides insight into how signaling the use of digital technologies may shape job seekers’ perceptions of organizational attractiveness. It supplements the employer attractiveness literature with empirical evidence on the role of technological signals in the digital age. Collectively, these insights connect signaling theory with employer attractiveness research more closely in the technological context and provide a more nuanced theoretical explanation for the formation of organizational attractiveness in the era of AI-driven recruitment.

Furthermore, existing research has adopted diverse theoretical perspectives to explain the various factors that influence organizational attractiveness during the recruitment process. For example, person–organization fit theory suggests that job seekers are attracted to organizations when they perceive congruence between their own values and goals and those of the organization (Firfiray & Mayo, 2017; Yu, 2014). The instrumental–symbolic framework indicates that job seekers are drawn not only to tangible organizational attributes but also to the symbolic meanings embedded in organizational image (Davies et al., 2025; F. Wu & Lei, 2026; Zimmer et al., 2026). Drawing on social identity theory, several studies demonstrate that organizations can boost their attractiveness by strengthening job seekers’ organizational identification (Liao & Cheng, 2020; Umrani et al., 2022; Younis & Hammad, 2021).

Although these theoretical perspectives have contributed to our understanding of the factors that enhance organizational attractiveness, they have largely focused on non-technological antecedents such as value congruence, organizational image, procedural fairness, and corporate social responsibility. This study extends these established frameworks by introducing AI-adoption signaling as a novel technological antecedent. In doing so, it addresses recent calls to incorporate AI-related characteristics into explanations of organizational attractiveness to job seekers (Basu et al., 2023; Black & van Esch, 2020; Chowdhury et al., 2023; Pereira et al., 2023).

Finally, this work finds that job seekers’ AI self-efficacy moderates the positive indirect association between AI-adoption signaling and organizational attractiveness via perceived innovation ability, highlighting the importance of receiver heterogeneity in signaling theory. Previous research on signals in recruitment materials has mostly focused on the design and transmission of organizational signals, with relatively limited exploration of how individual characteristics of signal receivers shape signal interpretation and response (Carpentier et al., 2019; Connelly et al., 2024; Highhouse et al., 2007; Iseke & Pull, 2019; Kakavand et al., 2022). This finding highlights the role of individual differences in interpreting technological signals. Theoretically, these findings contribute to signaling theory by suggesting that signal interpretation is contingent upon the receiver’s cognitive and experiential resources. Thus, individual differences play a critical role in the reception and interpretation of technological signals, confirming that both signal content and receiver characteristics interact in the signaling process. The effect of organizational signals depends not only on the signals themselves, but also on the receiver’s decoding skills and values.

### 7.2. Theoretical Contributions

This work’s theoretical contributions are as follows: First, this study conceptualizes AI-adoption signaling as a unique employer signal, transcending traditional recruitment cues such as salary, corporate culture, and corporate social responsibility (Aronson et al., 2025; Connelly et al., 2011, 2024; D. Phillips et al., 2024; Waples & Brachle, 2020). It systematically examines how AI-related signals influence organizational attractiveness, expanding signaling theory’s application within digital recruitment contexts. Furthermore, previous research has largely emphasized AI’s instrumental benefits in recruitment, such as improved recruitment efficiency (Basu et al., 2023; Black & van Esch, 2020; Chowdhury et al., 2023; Pan & Froese, 2023; Pereira et al., 2023); however, this study shifts the focus to how organizations’ AI-adoption signaling increases their attractiveness to potential applicants, thereby extending AI-related research.

Second, this study suggests a possible mediating mechanism linking AI-adoption signaling to organizational attractiveness, with perceived innovation ability as a mediator. This finding offers insights into how AI signals shape job seekers’ perceptions of organizational attractiveness, thereby contributing to our understanding of AI-related signals in recruitment. Moreover, this study found that perceived innovation ability is not only a general antecedent of employer attractiveness (Davies et al., 2025; Maier et al., 2022; Sommer et al., 2017) but also a technology-specific mediator that connects organizational digital signals to job seekers’ attraction evaluations. While existing research has established a correlation between perceived innovation ability and employer attractiveness, it lacks an explanation of how this perception functions as a transformative mechanism for technological signals (Davies et al., 2025; Maier et al., 2022; Sommer et al., 2017); our study provides evidence of its mediating role in the association between organizational AI-adoption signaling and employer attractiveness, thus refining its nomological status in the employer attractiveness literature. Revealing this mediating process establishes an important theoretical link between technological signals and talent attraction outcomes.

Third, this research suggests that potential job seekers’ AI self-efficacy moderates their interpretation of organizational AI signals. This finding highlights the critical role of individual differences in the reception of organizational signals and enhances the two-way interaction perspective of signal transmission, indicating that the impact of such signals depends on their content, as well as the recipients’ characteristics (Connelly et al., 2011, 2024; Highhouse et al., 1999; Spence, 1973). Moreover, this research introduces AI self-efficacy into the recruitment literature, thereby advancing the scholarship on AI self-efficacy (Dong et al., 2024; Kim & Lee, 2025).

Finally, this study contributes to the integration of AI technology application and organizational attractiveness. Although AI is increasingly being adopted in HRM, the academic exploration of AI’s influence on talent attraction remains nascent (Figueroa-Armijos et al., 2023; Veiga et al., 2023). By integrating the literature on AI-adoption signaling with studies of organizational attractiveness, this work not only empirically supports theoretical research on AI in HRM but also opens novel avenues for understanding employer signals in the digital age.

### 7.3. Practical Implications

Our study’s findings offer several practical implications. First, for organizations operating in China, strategically leveraging AI-adoption signaling in job advertisements carries substantial value. Contrary to a one-size-fits-all approach, our findings suggest that AI-adoption signals can effectively convey a progressive and innovative corporate image (Broekhuizen et al., 2023; Mariani et al., 2023; Roy et al., 2025), particularly resonating with job seekers in the Chinese market. This insight provides critical guidance for Chinese enterprises to design recruitment content that aligns with national digital transformation strategies, thereby enhancing organizational attractiveness and strategic talent acquisition capabilities.

Additionally, participants with higher AI self-efficacy levels were more attuned to signals of innovation related to AI adoption in China. This finding suggests that for Chinese organizations seeking to attract talent with strong AI-related capabilities, explicitly mentioning AI adoption in job postings could serve as an effective signal. Moreover, given that AI self-efficacy shapes how candidates interpret such signals, organizations might also consider including messages that emphasize support for employee adaptation to new technologies. Such an approach may help address potential concerns among candidates with lower AI self-efficacy, thereby enhancing organizational attractiveness among this group as well.

Finally, based on the heterogeneous characteristics across industries and job types, real-world recruitment contexts must not be oversimplified according to a one-size-fits-all logic. Job seekers’ perceptions of AI signals differ significantly; thus, business managers should adopt differentiated design strategies. For instance, job seekers in high-tech industries may exhibit high AI self-efficacy and prioritize an enterprise’s technological innovation capacity and development potential (Barba et al., 2025; Chen, 2024). Such enterprises should elaborate on their AI application practices in recruitment materials to strengthen candidates’ perceptions of the firm’s innovation capabilities and align with the career development needs of high-quality technical talent. In contrast, job seekers in traditional non-technical industries have lower AI self-efficacy, and their primary focus is on work efficiency and job stability. For this group, enterprises should frame AI-adoption signals around the core of “AI-driven work efficiency improvement” rather than emphasizing the technical sophistication of AI itself. In addition, candidates applying for technical roles also demonstrate high AI self-efficacy. Enterprises should explicitly link AI applications to career development opportunities in recruitment materials, thereby enhancing the alignment between such signals and candidates’ career expectations and boosting the firm’s attractiveness to technical talent. For non-technical positions, where job seekers have little demand for AI professional skills (Zhao et al., 2025), enterprises should highlight the indirect value that AI applications bring to these roles, helping candidates recognize the practical relevance of AI to their day-to-day work. By tailoring strategies to specific industrial attributes and job types, enterprises can effectively enhance the precision and practical value of AI-adoption signals in recruitment materials.

### 7.4. Limitations and Future Research

This study has several limitations. First, the experimental context involved simulated recruitment advertisements, and organizational attractiveness was primarily assessed based on job seekers’ subjective perceptions. Future research should use actual behavioral indicators, such as job application submissions, to measure organizational attractiveness more objectively. In Study 2, we enhanced our findings’ external validity and real-world applicability by recruiting actual job seekers to participate in our experiment (J. M. Phillips et al., 2023); nevertheless, future studies could further employ field experiments to examine how genuine recruitment messages influence actual job-seeking behavior.

Second, the strong correlation between perceived innovation ability and organizational attractiveness warrants careful interpretation. Our discriminant validity analyses indicated that the two constructs are empirically distinct and that a two-factor model is superior to a one-factor model. Nevertheless, we acknowledge that the study’s reliance on self-report, single-source data gives rise to the potential for common method bias (Podsakoff et al., 2012). Future research could employ temporal separation between measuring the mediator and the outcome or use objective behavioral outcomes (e.g., actual application intentions) to further mitigate potential concerns about conceptual overlap and common method bias.

Third, this study did not conduct a formal pretest to assess the realism and credibility of the job postings used in the experiments. Although the results of the manipulation check indicate that the AI signal was manipulated as intended, future research should employ more rigorous pretesting of experimental materials to better align them with real-world recruitment contexts. Field experiments using authentic recruitment information would further enhance the external validity of the findings.

Fourth, our data were collected exclusively from participants in China, limiting our findings’ generalizability. Given significant national and institutional differences in perceptions of AI adoption, the conclusions of this study have their external validity constrained by the study’s single-context design. Future research is urgently required to confirm this theoretical model’s generalizability through replication in cross-cultural contexts (J. M. Phillips et al., 2023). Specifically, future studies could examine whether job seekers from different cultural backgrounds perceive the innovative ability of organizations using AI differently and assess how these perceptions impact organizational attractiveness in distinct ways.

Finally, the experimental manipulation in this study focuses on the “AI-adoption signal” sent by organizations, rather than their actual level of AI adoption. Although this aligns with our theoretical focus, future research could further examine how job applicants respond when the signals sent by organizations conflict with their actual condition. In addition, whether the specific presentation of signals (e.g., detailed descriptions vs. brief mentions) affects their effectiveness also warrants further investigation.

## Figures and Tables

**Table 1:** Confirmatory factor analysis.

Models	χ^2^/df	RMSEA	CFI	TLI	SRMR
Four factor model: AI adoption signals, perceived innovation ability, organizational attractiveness, AI self-efficacy.	1.75	0.06	0.98	0.98	0.02
Three factor model: the combination of perceived innovation ability and organizational attractiveness	5.21	0.06	0.91	0.89	0.05
Three factor model: the combination of AI adoption signals and perceived innovation ability.	6.24	0.14	0.89	0.86	0.05
Three factor model: the combination of AI adoption signals and organizational attractiveness.	8.73	0.18	0.83	0.79	0.08
Two factor model: the combination of AI adoption signals, perceived innovation ability, organizational attractiveness.	10.62	0.20	0.78	0.74	0.08
One factor model: the combination of AI adoption signals, perceived innovation ability, organizational attractiveness, AI self-efficacy.	12.73	0.22	0.73	0.68	0.11

**Table 2:** The results of the regression analysis.

	Perceived Innovation Ability			Organizational Attractiveness	
	Model1	Model2	Model3	Model4	Model5
*Control variable*					
Gender	0.19 (0.20)	0.03 (0.14)	0.05 (0.13)	0.15 (0.17)	0.03 (0.15)
Age	−0.02 (0.03)	0.00 (0.02)	0.00 (0.02)	−0.01 (0.03)	0.00 (0.03)
Education	−0.29 (0.17)	−0.08 (0.12)	−0.13 (0.12)	−0.33 * (0.16)	−0.17 (0.13)
Work experience	0.04 (0.04)	0.02 (0.03)	0.02 (0.03)	0.02 (0.04)	0.00 (0.03)
*Independent variable*					
AI adoption signals		0.61 *** (0.04)	0.59 *** (0.04)		0.48 *** (0.04)
*Mediator*					
Perceived innovation ability					
*Moderator*					
AI self-efficacy			0.27 ** (0.10)		
AI adoption signals × AI self-efficacy			0.14 ** (0.04)		
R^2^	0.03	0.55	0.57	0.02	0.38
F	1.92	56.97 ***	43.87 ***	1.42	28.35 ***

Note: N = 240; * *p* < 0.05, ** *p* < 0.01, *** *p* < 0.001. The standard errors of the parameter estimates were indicated in parentheses.

## Data Availability

The data that support the findings of this study are available from the corresponding author upon reasonable request.

## Appendix A. Questionnaires

### Appendix A.1. Organizational AI Adoption (Cheng et al., 2023)

(1) This company has invested funds in AI technology.
(2) This company has been involved in the adoption of AI technology.
(3) This company has been interested in adopting AI technology to gain a competitive advantage.

### Appendix A.2. Perceived Innovation Ability (Luo & Bhattacharya, 2006)

(1) I believe that this company is highly innovative.
(2) In my view, this company demonstrates strong innovation.
(3) I am convinced that this company is at the forefront of innovation.

### Appendix A.3. Organizational Attractiveness (Highhouse et al., 2003)

(1) For me, this company would be a good place to work.
(2) I would not be interested in this company except as a last resort.
(3) This company is attractive to me as a place for employment.
(4) I am interested in learning more about this company.
(5) A job at this company is very appealing to me.

### Appendix A.4. AI Self-Efficacy (Dong et al., 2024)

(1) I am confident about my ability to do my job related to the AI/working with the AI.
(2) I am self-assured about my capabilities to perform my work activities related to the AI.
(3) I have mastered the skills necessary for my job related to the AI.
